# Factors Contributing to Exacerbating Vulnerabilities in Global Clinical Trials

**DOI:** 10.3389/fphar.2017.00999

**Published:** 2018-01-17

**Authors:** Ricardo E. da Silva, Angélica A. Amato, Dirce B. Guilhem, Marta R. de Carvalho, Elisangela da C. Lima, Maria Rita C. G. Novaes

**Affiliations:** ^1^Office of Clinical Trials, Brazilian Health Regulatory Agency (Anvisa), Brasília, Brazil; ^2^Faculty of Health Sciences, University of Brasília (UnB), Brasília, Brazil; ^3^School of Medicine, Health Sciences Education and Research Foundation (Fepecs), Brasília, Brazil; ^4^Faculty of Pharmacy, Federal University of Rio de Janeiro, Rio de Janeiro, Brazil

**Keywords:** clinical trials, vulnerable population, vulnerability, socioeconomic status

## Abstract

**Background:** Although policies and guidelines make use of the concept of vulnerability, few define it. The European Union's directive for clinical trials does not include explanations for or the reasoning behind the designation of certain groups as vulnerable. Emerging economies from lower middle-income countries have, in recent years, had the largest average annual growth rate, as well as increase, in number of clinical trials registered in the US government's database. Nevertheless, careful supervision of research activities has to be ensured.

**Objective:** To describe and analyze the features of the clinical trials involving vulnerable populations in various countries classified by development status and geographic region.

**Methods:** Retrospective study that involved analysis of data obtained from the International Clinical Trials Registry Platform (ICTRP) database between 01/2014 and 12/2014 from countries with (i) highest trial densities during 2005 to 2012, (ii) highest average growth rate in clinical trials, and (iii) greatest trial capabilities.

**Results:** Statistical analysis of this study showed that patients incapable of giving consent personally are 11.4 times more likely to be vulnerable patients than patients who are capable, and that patients in upper-middle-income countries are 1.7 times more likely to be vulnerable patients than patients from high-income countries when participating in global clinical trials. Malaysia (21%), Egypt (20%), Turkey (19%), Israel (18%), and Brazil (17%) had the highest percentages of vulnerable populations involving children.

**Conclusions:** Although the inability to provide consent personally was a factor associated with vulnerability, arbitrary criteria may have been considered when classifying the populations of clinical trials as vulnerable. The EU Clinical Trials Register should provide guidance regarding exactly what aspects or factors should be taken into account to frame given populations as vulnerable, because vulnerability is not applicable to all risk situations.

## Background

The increase in clinical trials carried out in developing countries raises concerns regarding careful supervision of research activities, protection of subjects' rights, and consent process integrity as well as attainment of valid scientific conclusions through populations with ethnic and cultural differences (Thiers et al., 2008). According to The World Medical Association Declaration of Helsinki (2013) some populations studied are particularly vulnerable, and thus require particular protection (The World Medical Association Declaration of Helsinki, 2013). The inclusion of vulnerable disadvantaged people in medical research is only justifiable if the research relates closely to these participants' health needs and priorities (Sengupta et al., 2010; Petrini, 2011).

One can define a person or group as “vulnerable” if they are relatively—or absolutely—unable to protect their own interests—perhaps because they lack the power, education, resources or other characteristics needed to do so. The term includes people who are unable to protect themselves against intimidation, threats, or the inappropriate use of influence. It is vulnerable groups which are most likely to experience abuse—examples being the chronically ill or incarcerated, the elderly, children, pregnant women, and populations in regions with fewer resources (Moreno and Arteaga, 2012).

Subjects with cognitive impairment are vulnerable to coercion, as they may have difficulty in making decisions based on evaluation of the possible risks and benefits of the study. Besides people with serious or potentially disabling or life-threatening conditions, others at risk of vulnerability are people living in nursing homes, recipients of welfare payments or social assistance, people in lower income brackets, the unemployed, patients in urgent or emergency care units, certain ethnic or racial minority groups, the homeless, members of nomadic communities, refugees, people displaced by conflicts or natural disasters, the incarcerated, the incurably ill, individuals from communities which lack political representation, and members of groups which are unfamiliar with the concepts of modern medicine (CIOMS, 2016).

For citizens of developing countries, being socio-economically disadvantaged may reduce their ability to consent freely. This is a form of vulnerability). In low and middle income countries, it is an easy matter to find patients willing to participate in clinical trials, as for many of these, enrolling in a clinical trial may be the only means of accessing health care (Weigmann, 2015). Engaging with communities has been recommended as a strategy with great potential for reducing inequalities in health (O'Mara-Eves et al., 2015).

Although the use and dimensions of the concept of vulnerability, and of the notion of a “vulnerable population,” have been much discussed, there remains an absence of consensus regarding the concept's meaning and application in research ethics. While some researchers believe that the term “vulnerability” is too broad and that it can result in the inclusion of unnecessary protection for some groups of individuals, others consider that some at-risk groups are not included in its scope and fail to receive the necessary protection. While many policies and guidelines make use of the concept of vulnerability, few succeed in defining it, preferring instead to discuss it in terms of the groups it describes. The lack of consistent normative status for the concept of vulnerability results in misunderstanding of such guidance as there is (Bracken-Roche et al., 2017).

Emerging economies, typically those of lower-middle income (LMI) countries, were found to have experienced the highest average annual growth rates between 2005 and 2012 (Drain et al., 2014). This rate corresponded to the annual number of clinical trials registered on the US government's Clinical Trials database. These countries can be competitive in attracting global clinical trials.

This study aimed to describe the features of the clinical trials involving vulnerable populations in various countries, classified by development status and geographic region; and to analyze factors which contribute to vulnerabilities in global clinical trials–such as country income, clinical condition, the patient's inability to provide consent, emergency situation, and studies involving children and adolescents.

## Methods

### Design

The study reported here was a cross-sectional overview. The data was obtained from the International Clinical Trials Registry Platform (ICTRP) database. The study focused on studies registered with the ICTRP between 01/01/2014 and 12/31/2014. The period of data collection was: 03/01/2014–06/31/2015.

Information was collected from the EU Clinical Trials Register (EUCTR), which has been a primary registry with the World Health Organization Registry Network since September 2011, and contains information on interventional clinical trials on medicines conducted in the European Union or European Economic Area (European Medicines Agency, 2016). This database was selected because it includes information on vulnerable populations. According to the glossary of terms used in the EU Clinical Trials Register (EU Clinical Trials Register, 2014), “specific vulnerable populations” means that among the participants in a clinical trial, one finds subjects—whether healthy volunteers or patients—who, it is considered, constitute an at-risk population. In the context of a study's inclusion in the EUCTR, however, it was unclear which criteria would be used by the study sponsor or by its delegate to consider a particular study population as vulnerable.

### Selection criteria

#### Inclusion criteria

Clinical trials registered in a primary registry (EUCTR) and ICTRP, that involved drug interventions in countries with highest average annual growth rates and trial density.

#### Exclusion criteria

**Figure 1:**
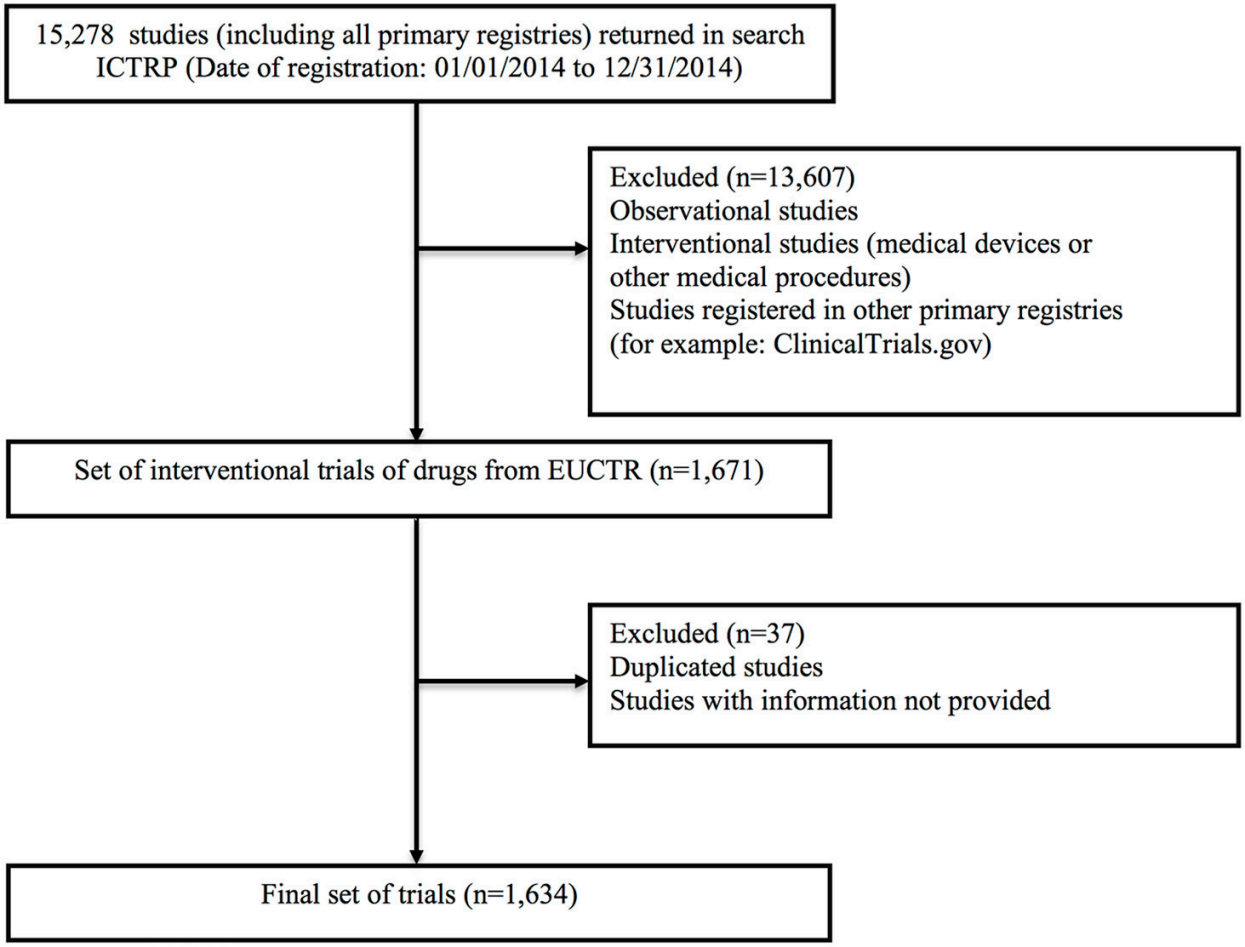
Study selection criteria. Adapted figure (Williams et al., 2015).

Observational studies, devices and medical procedures studies, and studies registered in other primary registries (for example: ClinicalTrials.gov) (Figure 1).

Clinical trials were selected from countries with (i) highest trial densities during 2005 and 2012, based on the average annual growth rate and trial density (annual number of registered clinical trials divided by country population in 2010) (Drain et al., 2014), (ii) highest average growth rate in clinical trials (Drain et al., 2014) and (iii) greatest trial capabilities (calculated as the mean number of clinical sites in each trial, contributed in large-scale trials in each country) (Thiers et al., 2008). This strategy led to the inclusion of clinical trials from Denmark, Estonia, Netherlands, Israel and Finland (criteria i), China, Egypt, India, Brazil, Turkey, Ukraine, Colombia, Singapore, Russia, Thailand, and Malaysia (criteria ii) and Argentina, Mexico, Chile and Peru (criteria iii) (Thiers et al., 2008; Drain et al., 2014).

#### Selected variables

Vulnerable populations; patients incapable of giving consent personally; emergency situations; health condition classified by International Classification of Diseases; age group, sponsor and development phase. The main variables (country income, clinical condition, patient's inability to provide consent, emergency situation and studies involving pediatric population and adolescents) were chosen for evaluation in the study, based on vulnerable groups identified in international research ethics guidelines and policies (Bracken-Roche et al., 2017).

All the steps for the search for the complete list of included clinical trials and their extracted characteristics are described below. This list can be seen as a file with supporting information.

Searching for studies on the platform, it is necessary to identify the search terms and to select filters. In the site (http://www.who.int/ictrp/en/), the type of search chosen was: advanced. In the fields (title, condition, intervention, primary sponsor and secondary ID) there was no use of search terms. All recruitment statuses and phases were selected. The search was performed for each country separately. The date of registration was 01/01/2014–12/31/2014. Only studies registered in EUCTR (interventional clinical trial of medicinal product) were selected. When accessing the primary registry link (www.clinicaltrialsregister.eu), “any country” was chosen, considering that it is the same trial protocol in all countries. The information was collected from the following fields: “B.1.1 (Name of Sponsor); E.1.1 (Medical condition being investigated); E.7 (Phase); F.1 (Age Range); F.3.3 (Specific vulnerable populations: Yes or No); F.3.3.5 (Emergency situation: Yes or No); F.3.3.6 (Subjects incapable of giving consent personally: Yes or No)”.

No bias control procedure was used. There was no calculation of the sample size.

#### Quantitative variables

The vast majority of these are international multicenter clinical trials; as trials frequently take place in multiple countries at the same time, studies may be duplicated by being registered in more than one country. However, considering that the analysis was performed by country, these duplicated studies cannot be excluded (Figure 1). To evaluate vulnerability, information was considered as a comparison of being vulnerable (yes) in relation to not being vulnerable (no). For each variable, the frequency of response between vulnerability and absence of vulnerability was verified. Later, the association between each variable and vulnerability was analyzed.

### Analysis

Study populations' ages were classified in accordance with the suggestions of the National Institute of Health. Age filters include: “80 and over: 80+ years; Aged: 65+ years; Middle Aged: 45–64 years; Adult: 19–44 years; Adolescent: 13–18 years; Child: 6–12 years; Preschool Child: 2–5 years; Infant: 1–23 months; Newborn: birth-1 month” (National Institute of Health, 2016). The pediatric population was considered as: studies that involved newborn and/or infant and/or preschool child and/or child.

Countries were classified according to economic development category as either “High-income” (HI), “Upper-middle income” (UMI), “Lower-middle income” (LMI), or “Low-income” (LI)–depending on how they were categorized by the World Bank (World Bank, 2016).

The World Bank classifies countries into four income groups. Economies were divided according to 2016 Gross National Income (GNI) per capita with income being categorized as: “(i) Low income: per capita GNI of US$1,025 or less (ii) Low-middle income: per capita GNI between US$1,026 and US$4,035 (iii) Upper-middle income: per capita GNI between US$4,036 and US$12,475 (iv) High-income: per capita GNI of US$12,476, and over.”

Countries were classified according to geographical regions (continental), based on their categorization by the United Nations (United Nations, 2016).

The study sponsor was classified according to the information on the organization's website. The WHO ICTRP defines the primary sponsor as the “organization which takes responsibility for the initiation, management and/or financing of a clinical trial” (World Health Organization, 2017a). Clinical condition was classified as incurable or not by a medical professional.

Predictors of vulnerability were identified using logistic regression model analysis, with a risk model being generated through the use of the strongest predictors. After verifying that the proposed regression model fitted well to the data, the Wald test was used. The association's strength was calculated for each of the variables. Where variables lacked a statistically-significant association (*p* < 0.05), they were removed from the final risk model. Odds ratios with confidence intervals of 95% were produced for the variables in the final risk profile model. No statistical method was used to control for confounding variables. No methods were used to consider the sampling strategy.

The study was approved by the Research Ethics Committee of the Health Sciences College of the-University of Brasília (Brazil).

## Results

All variables, except “emergency situation,” presented significant results in relation to being or not vulnerable (Table 1). The variable “emergency situation” did not present a statistically significant difference between being or not being vulnerable (*p* = 0.1857). One cannot, therefore, draw conclusions about emergency situation, regarding vulnerability. According to the results of the model and the value of the OR, it can be said that income is a risk factor for being, or not, vulnerable. It may also be observed being from a low middle income country is a risk factor for being, or not, vulnerable (in relation to being from a high-income class).

**Table 1:** Risk profile model for vulnerability, obtained by logistical regression.

**Type of variables**	**OR (95% CI)**	***P*-value**
Income (low middle vs. high)	1.4 (0.8–2.2)	<0.0001
Income (upper middle vs. high)	1.7 (1.3–2.3)	<0.0001
Unable to give consent	11.4 (5.3–24.4)	<0.0001
Pediatric population	1.6 (1.0–2.7)	0.0326
Adolescents	6.6 (3.7–11.5)	<0.0001
Emergency situation	1.9 (0.7–5.4)	0.1857
Incurable condition	1.4 (1–1.9)	0.0045
Transnational pharmac. I.	2.5 (1.9–3.2)	<0.0001

Patients from LMI countries have 1.4 more chance of being vulnerable patients than patients in HI countries. On the other hand, being a patient from a high-middle-income country is a risk factor for being, or not, vulnerable (in relation to being from a high-income class). Patients in UMI countries are 1.7 times more likely to be vulnerable patients than patients from HI countries.

The search in ICTRP returned 15,278 studies. After this, only studies from the EUCTR were selected (*n* = 1,671). A pivot table was created (dynamic table in Microsoft Excel 2016) based on a dynamic data source to match the data of the variables. Fifteen studies were excluded because they did not have information on emergency situations or patients' inability to provide consent or on the sponsor. Also, 22 studies were excluded because they were duplicated, that is, the same study was registered twice in the same country. After eliminating these studies, the database had 1,634 studies.

### Patients incapable of giving consent personally

According to the results of the model and the value of the odds ratio, it can be said that the inability to provide consent personally is a risk factor for being vulnerable or not. Patients who are unable to consent personally are 11.4 times more likely to be vulnerable patients than patients who are able.

### Pediatric population

According to the results of the model and the value of the odds ratio, it can be said that being part of a pediatric population is a risk factor for being vulnerable or not. Patients in the pediatric population are 1.6 times more likely to be vulnerable than those who are not.

### Adolescents

According to the results of the model and the value of the odds ratio, it can be said that being an adolescent is a risk factor for being vulnerable or not. Adolescent patients are 6.6 more likely to be vulnerable than non-adolescent patients.

### Incurable condition

According to the results of the model and the value of the odds ratio, it can be said that having an incurable clinical condition is a risk factor for being vulnerable or not. Patients who have incurable clinical conditions are 1.4 times more likely to be vulnerable patients than patients who do not.

### Transnational pharmaceutical industry (TPI)

According to the results of the model and the value of the odds ratio, it can be said that participating in a clinical trial sponsored by a TPI is a risk factor for the patient being vulnerable or not. Patients enrolled in a clinical trial sponsored by a TPI are 2.5 times more likely to be vulnerable than patients who are not.

**Figure 2:**
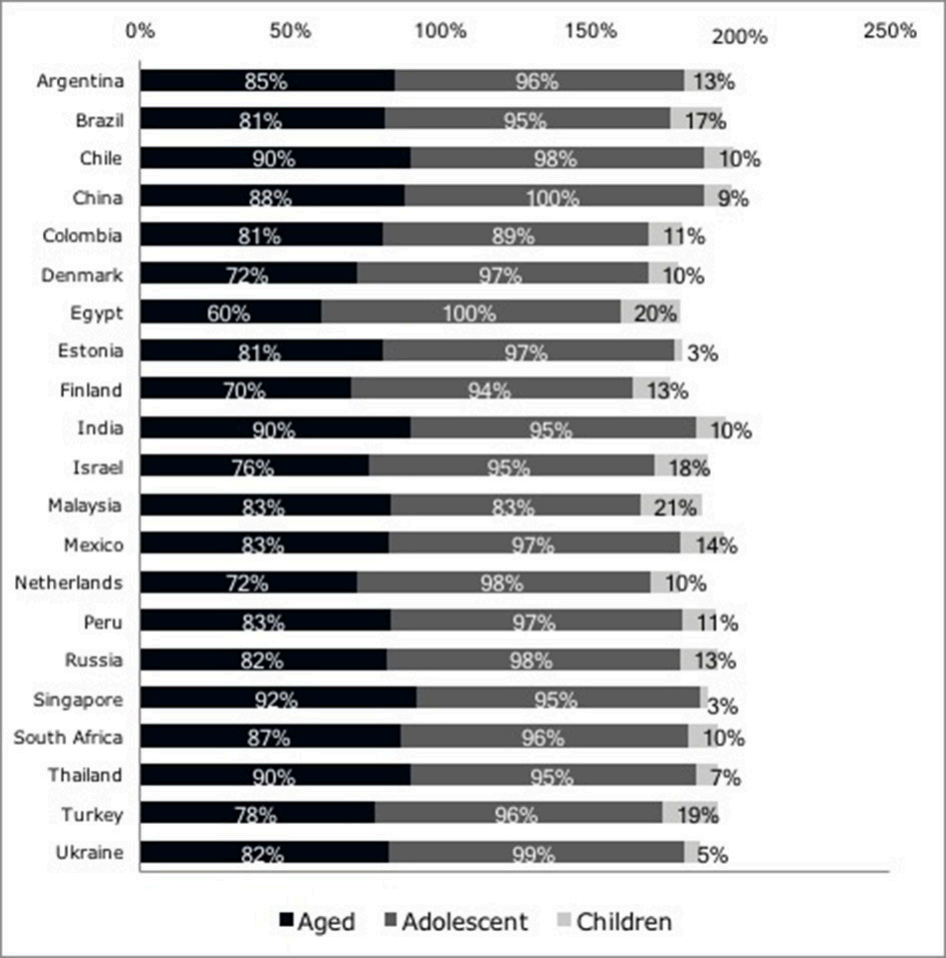
Percentage of clinical trials involving vulnerable populations by age group and country, the (EU Clinical Trials Register, 2014).

Clinical trials involving vulnerable populations are concentrated in Latin America and Asia (Table 2). Phase III studies involving vulnerable populations are the most prevalent (Table 3). Egypt, Malaysia and Turkey are countries with the highest percentage of children in clinical trials (Figure 2).

**Table 2:** Percentage of clinical trials involving vulnerable populations by countries, geographic region and development status, the (EU Clinical Trials Register, 2014).

**Country**	**Geographic region^a^**	**Development status^b^**	**% Studies involved vulnerable population**
Israel	Asia	HI	87
Turkey	Asia	UMI	87
Chile	L. America	HI	86
Brazil	L. America	UMI	84
Estonia	Europe	HI	84
Singapore	Asia	HI	84
Thailand	Asia	UMI	84
Russia	Europe	UMI	83
Argentina	L. America	UMI	83
South Africa	Africa	UMI	82
Mexico	L. America	UMI	82
Malaysia	Asia	UMI	80
Peru	L. America	UMI	80
Ukraine	Europe	LMI	80
China	Asia	UMI	79
Denmark	Europe	HI	75
India	Asia	LMI	74
Colombia	L. America	UMI	72
Egypt	Africa	LMI	63
Netherlands	Europe	HI	61
Finland	Europe	HI	59

a *United Nations (2016)*.

b *World Bank (2016)*.

*HI, High Income; LMI, Lower Middle Income; UMI, Upper Middle Income*.

**Table 3:** Percentage of clinical trials involving vulnerable populations by country and development phase, The (EU Clinical Trials Register, 2014).

**Country**	**Development phase (%)**								**Total**
	**I**	**I/II**	**II**	**I/III**	**II/III**	**III**	**III/IV**	**IV**	
Argentina	1	0	14	0	3	80	3	0	95
Brazil	3	0	13	2	0	75	5	3	76
Chile	2	0	12	2	2	80	2	0	58
China	3	3	15	0	6	70	0	3	42
Colombia	3	0	8	0	0	86	3	0	50
Denmark	1	3	37	0	0	37	2	20	168
Egypt	0	0	40	0	0	40	0	20	10
Estonia	0	3	11	3	6	72	0	6	43
Finland	0	21	0	0	0	64	2	13	80
India	0	0	5	5	5	85	0	0	27
Israel	0	1	23	1	2	69	4	0	95
Malaysia	0	0	13	0	4	75	4	4	30
Mexico	3	0	9	1	1	83	3	0	93
Netherlands	1	8	31	1	1	39	2	16	282
Peru	3	0	8	0	0	83	6	0	45
Russia	1	1	17	2	2	75	2	0	148
Singapore	0	11	24	0	3	57	0	5	44
South Africa	1	0	13	1	1	81	1	0	84
Thailand	2	5	12	0	3	71	0	7	49
Turkey	0	0	11	2	4	80	4	0	62
Ukraine	0	0	14	3	4	77	3	0	92

The diseases studied most in clinical trials were Chronic Obstructive Pulmonary Disease, Type 2 Diabetes Mellitus, Metastatic non-small cell lung cancer, Breast Cancer, Multiple Sclerosis and Homozygous Familial Hypercholesterolemia.

India, Singapore and Malaysia presented the highest percentages of clinical trials involving individuals incapable of providing informed consent.

## Discussion

According to the The World Medical Association Declaration of Helsinki (2013), certain research populations should be classed as vulnerable and in need of special protection, and the needs of people or communities which are considered to be disadvantaged economically or medically must be recognized. Most countries in this study were classified as upper middle income. Among the countries with the lowest percentages of vulnerable research populations, the following are classified as HI (Finland and Netherland); UMI (Colombia); LMI (Egypt and India). Despite this, as revealed in a series of articles published by the British newspaper “The Independent” in November 2011, multiple violations of patients' rights have taken place in the course of clinical trials in India (Jessop, 2012). On the other hand, countries such as India, Colombia, Egypt and Finland had the highest percentages of vulnerable populations unable to give informed consent personally. Although—for instance—Finland and Denmark are not characterized by major social, economic or health differences, specialists have observed increases in income inequality in these countries in recent years. However, one factor which could be more related to vulnerability could be the high prevalence of older adults in these high-income countries (Global Health and Aging, 2011; Canuto, 2012). In one study on cancer in children in Holland, it was observed that 25% of the parents of the children felt obliged to authorize the children's participation in the study. In another study, in the area of cardiology, which took place in Denmark, 18% of the participants stated that they had felt pressured to participate in the study (Mandava et al., 2012).

Legal incompetence, psychological inability, level of illiteracy and clinical conditions which stop the individual from communicating—such as a state of unconsciousness—all constitute aspects related to inability to provide informed consent. According to the results of the study, India, Singapore and Malaysia were the countries which presented the highest percentages of individuals incapable of providing informed consent. India continues to have high rates of illiteracy, in spite of advances in the last 10 years. In Singapore, parents' consent is required for those younger than 21 years old to participate in research (Rekhi et al., 2012; Kuthning and Hundt, 2013; United Nations Educational, Scientific, and Cultural Organization, 2017).

The developing countries have been associated with poor quality in the process of obtaining informed consent, due to aspects such as low educational levels, little understanding regarding what it means to participate in a scientific study, and difficulties in accessing the health services. However, one study undertaken in Brazil and the United States showed that even in a developed country, the participants' level of understanding regarding the studies can be deficient and can place these participants' well-being and rights at risk (Diemert et al., 2017). This may be related to the extent and complexity of the information contained in the informed consent form, as these are not always written in the simplest way, so that the participants will be able to understand them more easily (Krogstad et al., 2010). In the international multicenter clinical trials, a single model of the informed consent form may be used in all the countries involved in the study. This can be a problem, due to cultural, social, economic and educational differences between the regions. This document's translation to other languages should be reviewed to check for changes in meaning and content. The process of obtaining informed consent, therefore, must be carefully adapted to each country (Krogstad et al., 2010; Tamariz et al., 2013).

Despite remarkable progress in the reduction of poverty and inequality in Latin America in recent years, 92 million people there are classified as extremely poor, and 77 million as moderately poor. One can class a person as vulnerable if their daily income is between US$4 and US$10—which has been empirically observed to indicate a probability of falling into poverty that is above 10%. The incidence of vulnerable people in 2013 was: Brazil (38.4%), Colombia (36.7%), Argentina (34.4%), Chile (37.7%), Mexico (37.8%), Peru (40.5%), Venezuela (45.9%), and Ecuador (42%) (Stampini et al., 2015). This present study showed that Brazil and Chile are among those with the highest percentages of clinical trials involving vulnerable populations. At the time of writing, phase I clinical studies are not migrating to LMI countries, but it may be that in the future this situation will change. Phase I studies have been related to an ethical dilemma posed by the participation of economically or educationally disadvantaged healthy volunteers. The dilemma relates to the extent to which phase I studies—through the use of financial incentives—cause people on low incomes to be represented disproportionately in exploratory research. It is common for paid volunteers not to be able to access many treatments developed through clinical research, because they lack health insurance (Dresser, 2009; Iltis, 2009). Clinical trials involve risks to participants' health, as, at the time of the study, the safety—and side effects—of the drugs being tested are neither fully known nor fully understood. Accordingly, all participants are at risk when they are included in a trial (Weigmann, 2015). The definition of vulnerable population from the EU Clinical Trials Register is not useful, because it considers that vulnerable subjects are those that are at risk. It may be that the EU Clinical Trials Register's definition of vulnerable population lacks clarity because the EU Clinical Trials Directive and EU Clinical Trials Regulation do not discuss vulnerability in accordance with ethical principles and do not provide explanations regarding why certain identified groups are vulnerable (Bracken-Roche et al., 2017).

Any useful definition of vulnerability has to be sufficiently comprehensive to cover those requiring protection beyond what is considered normal, without including those for whom these additional levels of protection would unnecessary (Bracken-Roche et al., 2017). According to one study, of the 11 policies and guidelines evaluated, only the CIOMS guidelines and the Tri-Council Policy Statement (TCPS2), met the criteria for a full conceptualization of vulnerability (Bracken-Roche et al., 2017). The definition of vulnerability found in the TCPS2 is: “A diminished ability to fully safeguard one's own interests in the context of a specific research project. This may be caused by limited decision-making capacity or limited access to social goods, such as rights, opportunities and power.” The definition from TCPS2 seems to be the most useful, because it involves the principles of Respect for Persons, Concern for Welfare, and Justice. In addition, TCPS2 incorporates the issue of conflicts of interest in vulnerability (Canadian Institutes of Health Research, 2014).

Although several definitions have been elaborated for the term “vulnerable population,” uniformity and equitable standards are missing in the understanding and grading of risks posed to vulnerable populations globally. This impacts on acceptability and on the evaluation of risks which is needed in establishing consistent safeguards in biomedical research. As a result, understanding the factors which lead to vulnerability is important for planning the risk monitoring of clinical trials involving these populations (Shivayogi, 2013).

Although the inability to provide consent personally, which was an important factor related to vulnerability identified in this study, is a fundamental point to be used in the definition of vulnerable populations no correlation was found between emergency situations and vulnerability. While it is true that it is not possible to know of the types of emergency situation which occurred, that such situations appear not to be associated with vulnerability seems counter-intuitive. It cannot be ruled out that arbitrary criteria may have been used in classifying populations as vulnerable. Two possible reasons may be related to the fact that the “emergency situation” factor did not present a significant risk in relation to vulnerability: (i) the fact that it may not have been considered as a possible vulnerability factor by those responsible for registering the clinical trial in the EU Clinical Trials Register and (ii) because of the small number of clinical trials which involved emergency situations.

Vulnerable populations' right to improve their health through the use of research-based treatments does not decrease the necessity to ensure that studies should be of high quality and comply with restrictions regarding research in which these populations participate. Notwithstanding, vulnerable participants' access to clinical trials and to the benefits resulting from those trials must be ensured (Helmchen et al., 2014). The processes of any given society, whether social, economic or political, tend to reinforce social exclusion, discrimination and prejudice. In so doing, they bolster inequality–both in outcomes and in opportunities (United Nations Development Programme, 2013). It follows that access to participation in clinical trials must be guaranteed to vulnerable individuals (Kuthning and Hundt, 2013).

Vulnerable individuals' inclusion in research depends on the adequate functioning of the research ethics committees, which must have trained staff, investment and sufficient time to assess the research protocols. These committees must be able to identify those who are vulnerable and ensure measures such as the participation of a legal representative in the process of gaining informed consent when—for example—the participant is illiterate (Krogstad et al., 2010).

According to the principles of bioethics, every individual has the right to refuse to participate in a study without their current treatment being compromised. According to the study's results, Israel is one of the countries with the highest percentages of clinical trials involving vulnerable populations. Although it is not possible to identify which criteria were used for classifying these populations as vulnerable, there is a law in Israel which establishes that, under specified circumstances, a competent patient who is capable of taking decisions may receive forced medical treatment, even if he or she has refused this. For some specialists, this harms the patient's autonomy. Others, however, believe that respecting the patient's decision not to receive treatment may cause harm, when, in the physician's opinion, it is known that the treatment would significantly improve the patient's health (Gross, 2005).

The countries from Africa and Asia (Egypt, Israel, Malaysia, and Turkey) had the highest percentages of vulnerable populations involving children (Figure 2). A point to be considered is that children from countries in Africa and Asia are more likely to have serious conditions and mortality is much higher. For example, the chance for a child in Africa to die before age 5 is fifteen times higher than children in high-income countries. This may be a factor related to the inclusion of these children in clinical trials (The United Nations Inter-Agency Group for Child Mortality Estimation, 2017; World Health Organization, 2017b). Also, this percentage may be related to research incentives in this population or issues related to vulnerability, such as obtaining financial benefits in exchange for participation. Pediatric research incentives have increased the number of clinical trials involving children in the United States and EU (European Medicines Agency, 2012). Children are considered vulnerable as research subjects because, as their developmental stage affects their cognitive abilities, they are relatively unlikely to be able to assess the implications of participating in a study-or to appreciate the risks and benefits which participation may bring.

According to the guidelines of the International Conference on Harmonization (ICH), when the participant is not capable of providing informed consent—as in the case of illiterate patients—it is necessary for a legally accepted representative to participate in the process and provide this consent. In poor regions, however, the children—or even the adults—have difficulty in finding a legal representative. In these cases, the local ethics committee must decide how the consent is to be obtained (Cheah and Parker, 2014; ICH, 2016).

According to the results of the present study, Turkey is among the countries with the highest percentage of children in clinical trials. In this country, in the event of planning a trial involving children as research subjects, the 31/3/2005–5328/7 amended article of the Turkish Penal Code should be taken into consideration. Children's participation as research subjects is only permitted if the scientific data to be obtained makes this absolutely necessary. Besides the consent of any child considered able to provide this, researchers must also obtain consent from parents or legal guardians. Moreover, authorized committees to which research proposals are submitted must include pediatricians (Ilbars, 2013).

The EU Clinical Trials Register is a database containing important information; however, in relation to vulnerable populations, in particular, to information regarding whether the study population is vulnerable or not, it is not known exactly what aspects were taken into account in the framing of given populations as vulnerable when the studies were registered in the EU Clinical Trials Register. The definition of vulnerability by the European Union is not clear and the scope of the concept of vulnerability, as defined by the EU Clinical Trials Register, is broad. Vulnerability may, for instance, refer to social and economic disadvantages, restricted access to health services, or conditions associated with decreased decision-making capacity regarding participation in studies (Rivera et al., 2009). It is time that the EU Clinical Trials Directive and EU Clinical Trials Regulation re-considered the concept of vulnerability, based on the three principles mentioned above: Respect for Persons, Concern for Welfare, and Justice.

## Limitations

Countries from regions of Asia, Africa and Latin America may not have the habit of registering clinical trials in a European database. This may, therefore, impact on the results found in the present study, because other studies undertaken in Asia, Africa and Latin America may not be taken into account in the analysis, as only studies from the European register were analyzed.

Clinical trials registration is related to limitations such as poor quality of registered trial data; many clinical trials on the registers are incomplete, inaccurate, out-of-date or registered retrospectively.

Primary Registries found in the WHO Registry Network are expected to meet specific criteria regarding their content, quality, validity, accessibility, administration, unique identification and technical capacity. Primary Registries must adhere to the requirements of the International Committee of Medical Journal Editors. Data Providers are held to be responsible for any database used by one or more registries. The ICTRP accepts trial records only if it believes that these were set up and managed in a form consistent with the WHO Registry Criteria. In the South American region, only the Brazilian Clinical Trials Registry and the Peruvian Clinical Trials Registry are data providers. Therefore, the WHO trial registry cannot contain all trials held in the South American region, which may compromise the study of the current scenario of clinical trials in this region (World Health Organization, 2017c).

At the time of writing, the ICTRP accepts data from a total of 16 national and regional registries from various countries that meet their quality criteria (World Health Organization, 2017c). There are, however, other clinical trial registries, such as those developed by the pharmaceutical industry; as a result, this study may not have captured all clinical trials registered globally. The fact remains, however, that trial registration in WHO-approved registries is broadly endorsed.

Elderly (≥65 years) and pregnant women were variables excluded in this study. Despite elderly people and pregnant women having a vulnerable biological condition, they may not be as vulnerable to coercion or undue influence as children, who often cannot appreciate the implications of participating in research, nor adequately balance the risks and benefits posed by the research procedures (European Medicines Agency, 2012; Bracken-Roche et al., 2017).

No bias control procedure was used. The data collected were restricted to the clinical trials registered in the ICTRP in the period of 2014.

## Conclusions

Although inability to provide consent personally has been a factor associated with vulnerability, arbitrary criteria may have been considered in the classification of the clinical trials' populations as vulnerable. This is because “emergency situations” were not associated with vulnerability. It follows that the classification of the clinical trials' populations may be being undertaken arbitrarily, which may be related to the lack of clarity in the guidance regarding the concept and scope of vulnerability in the EU Clinical Trials Register and other related guides.

The understanding of what constitutes a vulnerable population may differ between countries, and different factors, concepts or scopes of vulnerability may have been considered by the study sponsors when they framed the participants as vulnerable. Therefore, the EU Clinical Trials Register should provide guidance regarding exactly what aspects or factors should be taken into account to frame given populations as vulnerable. Vulnerability is not applicable to all situations. However, patients may require enhanced protection of their rights if they are pediatric patients, unable to give consent, on low incomes or in certain clinical conditions. Further studies are necessary to improve the investigation of the criteria which are taken into account for classifying populations as vulnerable in the electronic register of clinical trials.

Among the five countries with the highest percentages of clinical trials involving vulnerable populations are those of Latin America. This may be related to socioeconomic inequalities.

Clinical trials involving children are still a minority and countries need to promote public policies for the development of drugs for this population. The highest percentages of studies involving children, among the selected countries, are in Asia. While it is necessary for countries to create incentives for conducting studies in the pediatric population, it is also necessary to guarantee this population's rights and well-being, as it is considered vulnerable.

## Author contributions

RS, MC, MRN and EL made substantial contributions to conception, design and acquisition of data. In addition to they analyzed and interpreted the data. AA and DG made substantial contributions to conception and design. All authors read and approved the final manuscript.

### Conflict of interest statement

The authors declare that the research was conducted in the absence of any commercial or financial relationships that could be construed as a potential conflict of interest.
